# Off-label applications of omalizumab: Current insights and perspectives^☆^

**DOI:** 10.1016/j.waojou.2025.101156

**Published:** 2025-12-12

**Authors:** Aleksandra Jaromin, Aleksandra Wardzyńska

**Affiliations:** Department of Immunology and Allergy, Medical University of Lodz, Poland

**Keywords:** Omalizumab, Food allergy, Off-label, SCIT, Drug hypersensitivity

## Abstract

Omalizumab is a humanized monoclonal antibody that binds free IgE. Consequently, it exhibits inhibitory properties against allergic cascades. Over the past 20 years, omalizumab has been in the market, and studies have shown its strong tolerability and safety profile. Since 2003 in the United States of America and 2005 in Europe, omalizumab has been available to patients as a therapeutic option. In Europe, it is registered for the treatment of allergic asthma, chronic spontaneous urticaria, and chronic sinusitis with polyps. In the United States, it has been registered for the treatment of allergic asthma, chronic spontaneous urticaria, and chronic food allergies in children over 1 year of age. With a universal target point for most allergic conditions, omalizumab has the potential to become the most versatile biological drug for allergologists. The literature describes numerous uses of omalizumab as an adjuvant or monotherapy for allergic conjunctivitis, systemic mastocytosis, food allergies, drug hypersensitivity, allergic bronchopulmonary aspergillosis, and allergen immunotherapy, among others. In the following publication, we will provide you with the current knowledge regarding the use of omalizumab in conditions other than those covered by the current product registration.

## Introduction

In 2005, omalizumab (OMA) was approved for marketing in the European pharmaceutical market. It is an antibody against IgE that binds immunoglobulins of this class and prevents them from binding to specific receptors, which then reduces the expression of receptors on the effector cells. This results in inhibition of the allergic cascade and IgE-mediated inflammation. Initially, OMA was used in the treatment of moderate or severe asthma, and over time, it was added to the indication for the treatment of chronic spontaneous urticaria (since 2014) and chronic rhinosinusitis with polyps (since 2020). Since February 2024 in the United States of America, omalizumab can also be used to treat food allergies in adults and children as young as 1 year of age. Over more than 2 decades on the market, the safety of OMA has been proven, in real-life studies, and when used, the most common side effects include mild and usually self-limiting local reactions. The risk of anaphylaxis and other serious side effects was relatively low.^1^ The incidence of cardiovascular events, cancer, infection, and impact on pregnancy has not been determined, especially in the long term (currently, the longest observations last about 20 years). Observing the dynamic development of immunology and allergology, it seems that this drug, which has a universal target point, may also be effective in other diseases involving IgE. The following publication summarizes the current progress in research on the use of omalizumab beyond its registered indications in Europe.

## Mechanisms of action of omalizumab

**Fig. 1:**
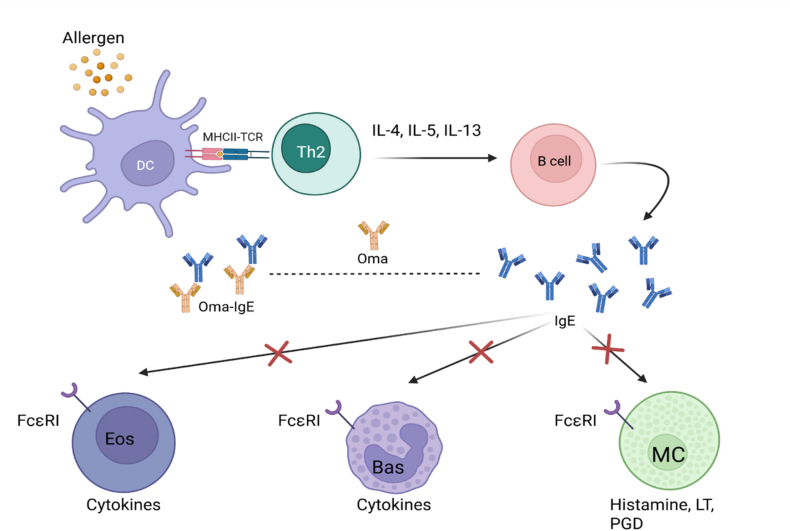
Omalizumab - influence on allergic reaction. Dendritic cells (DC) present antigens (allergens) to Th2 lymphocytes through interleukins, activate B cells, and initiate the production of allergen-specific IgE. Omalizumab (Oma) binds free IgE and prevents its binding to dedicated FcεRI receptors on eosinophils (Eos), basophils (Bas), and mast cells (MC), thus preventing the release of cytokines, histamine, leukotrienes, and prostaglandins

Omalizumab is a humanized recombinant IgG1 class monoclonal antibody that binds to circulating immunoglobulin E (IgE). It attaches to the C3 domain of IgE, which causes these antibodies to lose their ability to bind to specific receptors on the effector cells. Subsequently, the expression of FcεRI is secondarily reduced, mainly on mast cells, basophils, dendritic cells,^2^ and FcεRII, inter alia, on B lymphocytes.^3^ This leads to inhibition of Th2-mediated inflammation (Fig. 1).

**Fig. 2:**
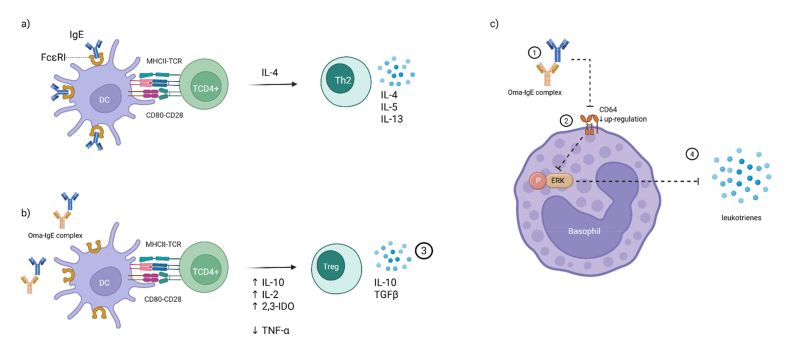
Influence of omalizumab therapy on immune cells. a) and b) The omalizumabe-IgE complex changes the cytokine profile and influences dendritic cells (DC) to differentiate naive TCD4+ lymphocytes into T regulatory lymphocytes (Tregs) rather than into T helper 2 lymphocytes (Th2). c) The omalizumab-IgE complex inhibits CD64 upregulation, thus preventing phosphorylation of extracellular signal-regulated kinases (ERK), thus enabling the release of leukotrienes

However, it seems that the mechanisms determining the efficacy of omalizumab are broader than initially assumed, and it has a much more extensive immunomodulatory effect by acting on regulatory T cells.^4^ It has been suggested that during the immune response to the allergen, the binding of IgE to the FcεRI receptor on the surface of dendritic cells causes, among other actions, inhibition of the production of regulatory T lymphocytes (Tregs), and the promotion of Th2-dependent inflammatory mechanisms. *In vitro* research has indicated that the use of OMA restores the ability of dendritic cells to stimulate the generation of FOXP3+ Tregs. This effect may depend on the effect of OMA on the production of cytokines that promote the polarization of T lymphocytes towards Tregs, such as IFN-α, IL-10, IL-2, and indolamine 2,3-dioxygenase (IDO), while reducing the concentration of TNF-α, which, under physiological conditions, inhibits the formation of regulatory lymphocytes^5^ (Fig. 2). These observations confirmed the IgE-independent mechanism of action of OMA in suppressing the immune response.

The multidirectional effect of omalizumab has also been considered in other studies using both cell lines and donor basophils. OMA not only blocks the binding of IgE to specific receptors, but also causes the dissociation of previously bound IgE from FcεRI. In addition, because IgE monomers are a vital factor in mast cells and basophils, OMA indirectly affects the viability of these cells. At the intracellular signaling level, OMA is known to reduce the phosphorylation of signaling proteins such as Syk, PLCγ, LAT, and ERK in mast cells, leading to a reduction in cell activation. This results in the reduced synthesis of lipid mediators, including pro-inflammatory leukotrienes (Fig. 2). Inhibition of early and late phosphorylation mechanisms enables the rapid action of OMA, even as early as 24 h after drug administration, which is most often seen in treatment of chronic urticaria.^6^

Omalizumab may also affect the formation of a subpopulation of T cells. In a study of patients with chronic spontaneous urticaria on OMA, a reduction in the number of both naïve CD4^+^ and CD8^+^ T cells was found in favor of an increase in memory CD4^+^ T cells and effector CD8^+^ T cell populations. An increase in the percentages of Th1 and Th2 lymphocytes was observed. Therefore, the authors of this study suggest that analysis of the T lymphocyte population may serve as a marker of an effective immune response to OMA.^7^

When considering the potential mechanisms of action of omalizumab as an adjuvant of immunotherapy, it has been pointed out that it is not only able to form biologically inactive complexes with IgE, but also has a modulatory effect on T lymphocyte subpopulations, reduces effector cell reactivity, and affects the efficiency of antigen presentation by APC cells or shifts in the concentrations of individual classes of antibodies.^8^

In a study analyzing changes in the immune response during oral immunotherapy (OIT) to ≥ 2 foods,^9^ changes in T-cell subpopulations have already been demonstrated during the omalizumab treatment phase prior to the initiation of OIT. At this stage, there was a decrease in the proportion of allergen-specific CD4^+^ T cells secreting IL-4, GPR15 expression on CD8^+^ and Th2 cells with a phenotype of effector memory cells (EM), and CXCR3-positive γδ and CD8^+^ EM cells. Both receptors are responsible for the migration of cells, especially T lymphocytes, to sites of inflammation. In week 36 of the study, further changes were observed in the expression of receptors associated with skin migration (CCR4 and CLA) on T cells. In addition, a decrease in the expression of costimulatory molecules (CD86) on APCs was observed, and the IgG4/IgE ratio for the allergen components increased significantly. In addition, there was a decrease in basophil activation and in the levels of cytokines, not only of the Th2 profile, but also of pro-inflammatory cytokines. Overall, these changes indicated a shift from the Th2-dominated allergic response and a reduction in the overall inflammatory markers following treatment with both immunotherapy and omalizumab.

## Selected off-label uses of omalizumab

### Allergen immunotherapy

Specific immunotherapy is an effective tool for reducing allergy symptoms when exposed to an allergen as well as altering the natural history of the disease in some cases.^10^ Currently, there are various types of immunotherapy (subcutaneous, sublingual, and oral) that target various groups of allergens, including airborne allergens (eg, tree pollen, grass pollen, house dust mites) and Hymenoptera venoms (wasps and bees), as well as food allergens. Regardless of the form of immunotherapy, it is effective in reducing the symptoms.^11^ A complete course of immunotherapy, usually lasting 3–5 years, can provide a reduction in symptoms for a long time, even after treatment is stopped. However, increased side effects, including asthma exacerbation and anaphylactic shock, may prevent the initiation or continuation of immunotherapy. In exceptional cases, when immunotherapy is necessary to improve quality of life, side effects are not sufficiently controlled with conventional medications, or there is a high risk of anaphylaxis, the use of OMA as adjuvant therapy may be helpful.

### Aeroallergens

Currently available data indicate that omalizumab is highly effective as an adjuvant for aeroallergen immunotherapy, and when used as monotherapy, it may have a prophylactic effect. In patients with asthma, it provides a protective effect against exacerbations of the disease and reduces the use of inhaled glucocorticosteroids (ICS) during immunotherapy. In contrast, in patients with allergic rhinitis undergoing immunotherapy, the severity of adverse effects is reduced.

According to a Polish real-life study in children with severe allergic asthma, the introduction of omalizumab before specific immunotherapy (SIT) prevented disease progression and reduced the incidence of asthma exacerbations.^12^

The use of OMA is primarily considered in cases of severe adverse effects of immunotherapy, co-morbidity with asthma, and/or severe chronic rhinitis. Omalizumab may be an alternative to allergen immunotherapy in the case of polyvalent allergies or when there is no dedicated preparation for specific immunotherapy.^13^ A summary of the studies on this application is presented in Table 1.

**Table 1:** The use of omalizumab during specific immunotherapy with airborne allergens.

Authors	Type of immunotherapy, allergen	Number of subjects	Type of study	OMA Dosage Schedule	Conclusions
Bożek et al.^54^	S.L. mites	N = 9	Observation	OMA used concurrently with SLIT for 24 months	In the OMA + SLIT group: TRSS, TMS and CTS scores improvement after 2 years.
Bożek et al.^55^	S.C. mites	N = 82	Double-blind, randomized, multicenter	OMA or OMA + SCIT or SCIT or placebo for 24 months	The most effective combination therapy OMA + SCIT; Less ICS consumption and fewer asthma exacerbations.
Stelmach et al.^12^	S.C. mites or molds	N = 17	Observation	OMA used in parallel with IT for 52 weeks	Fewer asthma exacerbations and hospitalizations due to asthma exacerbations and reduced ICS consumption.
Massanari et al.^56^	S.C. mite/dog/cat (at least 1 of the above)	N = 225	Double-blind, randomized, multicenter	16 weeks OMA or placebo course, at 13 weeks IT incorporation.	Better asthma control, higher likelihood of achieving IT maintenance dose, less frequent anaphylactic reactions.
Zhang et al.^57^	S.C various	N = 901	Meta-analysis	OMA + AIT or AIT	Increased efficacy and safety of AIT, higher likelihood of achieving maintenance dose and SU.

Abbreviations: s.l., sublingual; OMA, omalizumab; IT, immunotherapy; SLIT, sublingual immunotherapy; TRSS, total rhinitis symptom score; TMS, total medication score; CTS, combined total score (sum of TRSS and TMS); s.c., subcutaneous; SCIT, subcutaneous immunotherapy; ICS, inhaled corticosteroids; AIT, allergen immunotherapy; SU, sustained unresponsiveness

### Venoms of hymenoptera insects

In the case of insect venom allergy, case reports and case series are mainly available for patients who have received venom immunotherapy (VIT) using accelerated (RUSH) or ultra-accelerated (ultra-RUSH) protocols together with omalizumab. A pooled analysis of available studies indicates that OMA therapy should continue until the maintenance dose of VIT is reached or even 3–6 months after the maintenance dose is reached.^14^ The use of OMA in VIT should be considered in the following cases: the occurrence of intensified adverse effects during dose escalation, especially in the case of a history of severe anaphylaxis after a sting, in the pediatric population due to the reduction of adverse reactions, in the case of concomitant systemic mastocytosis, and in accelerated protocols, that is, RUSH and ultra-RUSH in patients with an increased risk of adverse reactions. The study data suggest that OMA should be continued until tolerance to VIT and stings has been achieved. VIT alone, during which omalizumab is used, should be continued for 3–5 years as standard.

In the European consensus on the treatment of Hymenoptera venom allergy in 2023, the authors propose to consider adding omalizumab to VIT if recurrent systemic reactions occur during VIT (despite eliminating the contributing factors and increasing the maintenance dose of VIT and antihistamines). According to the German consensus, in addition, if the maintenance dose of VIT does not appear to provide adequate protection against severe anaphylactic reactions, the addition of omalizumab to VIT for the duration of hymenoptera activity (from spring to autumn) may be considered.^15^ Please refer to Table 2 for a selection of studies on this topic.

**Table 2:** The use of omalizumab during specific immunotherapy with hymenoptera venom allergens.

Authors	VIT type, allergen	Number of subjects	Type of study	OMA Dosage Schedule	Conclusions
Gülsen et al.^58^	s.c., Apis mellifera	N = 1 (60F)	Case report	OMA 150 mg for 5, 3 and 1 week before the IT resumption and ongoing for the next 2 months.	OMA premedication allowed the continuation of VIT despite a previous episode of anaphylaxis in contact with the bee venom vaccine.
González-de-Olano D et al.^59^	s.c., Apis mellifera	N = 1 (32 M)	Case report	SAE after 7th dose of VIT; OMA 300 mg every 4 weeks, after 2 months of VIT recurrence. OMA continued for 12 months. After 9 months of the last dose of OMA, a sting test.	OMA enabled the continuation of immunotherapy against bee venom (despite the accompanying mastocytosis). The sting test performed after more than 3 years of VIT came out negative.
Stretz E et al.^14^	s.c., Apis mellifera, Vespula	N = 15	Case series	OMA at a dose of weight and IgE administered 5, 3 and 1 week before VIT recurrence. Discontinuation of OMA 3–6 months after achieving an increased maintenance dose (200–300 μg).	Temporary addition of OMA to VIT enabled tolerability of the increased maintenance dose of VIT and tolerance of VIT was maintained even after discontinuation of OMA (follow-up to 8,6 years).
Droitcourt C. et al.^60^	s.c., Apis mellifera	N = 3 (teenagers)	Case series	OMA at a dose of weight and IgE level, administered 4 and 2 weeks before VIT.	Brief OMA therapy (2 drug administrations) prior to resumption of VIT enabled tolerance of VIT in the RUSH protocol in subjects previously experiencing SAR during VIT. The tolerance of VIT was maintained for 4–5 years.

Abbreviations: s.c., subcutaneous; F, female; OMA, omalizumab; IT, immunotherapy; VIT, venom immunotherapy; M, male; SAE, serious adverse event; RUSH, accelerated; SAR, serious adverse reaction.

### Food allergens

Standard treatment options for food hypersensitivity beyond the treatment of acute exposure-related symptoms include long-term avoidance of the allergen and, in warranted cases, desensitization. The effectiveness of these interventions has been proven; however, food desensitization carries a certain risk of adverse reactions, including anaphylaxis. Omalizumab can be used as an adjuvant in mono- or multicomponent (for several foods at once) immunotherapy for food allergy. Selected studies have proven that it significantly increases the safety of immunotherapy (fewer serious adverse events have been demonstrated) and its use reduces side effects that may occur during OIT, such as itching of the mouth or throat as well as abdominal pain.^8^^,^^16^

In real-life study among patients with moderate to severe asthma and food-related anaphylaxis history omalizumab allowed reintroduction of allergenic foods to their diet (peanut, tree nuts, fish, egg, milk, and/or wheat) and improved ACT (Asthma Control Test) as well as Quality of life (QoL).^17^ An extension of OUtMATCH (Omalizumab as Monotherapy and as Adjunct Therapy to Multi-Allergen OIT in Food Allergic Participants) study also reported the possibility of reintroduction retail allergenic foods after omalizumab treatment (best results for milk, eggs and wheat) although adverse reactions still occurred and many participants returned to avoidance of allergenic food.^18^

A meta-analysis of 36 studies involving 953 patients investigated the efficacy and safety of OMA for food allergies. In terms of safety, omalizumab alone was not associated with serious adverse events. During the use of OMA, mild adverse events, such as reactions at the site of administration, occur.^8^^,^^19^ Among the potential benefits mentioned is the ability to achieve a maintenance dose in a shorter time, which means faster protection against anaphylaxis associated with accidental ingestion, as well as fewer visits to medical facilities. The authors of the publication drew the following conclusions: OMA allowed for faster and more effective desensitization of food, reduced the risk of anaphylaxis, and improved the quality of life of patients and their caregivers.^8^

According to the newest meta-analysis by Numatov et al based on controlled trials only, similar findings were made. Omalizumab improved desensitization rates and increased food tolerance thresholds as well as reduced the risk of allergic reactions related to food. Adverse or severe reactions (including skin reactions) were not increased. Another finding was that omalizumab caused decreased hypersensitivity and a lowered allergic and inflammatory response in immunologic system. No studies mentioned in the above meta-analysis evaluated cost-effectiveness.^20^

In summary, the results of the above meta-analyses showed that adding OMA to immunotherapy allowed for faster desensitization in a shorter amount of time. The treatment remained effective when a larger variety of foods were included in immunotherapy. OMA provides faster and more effective immunotherapy and reduces the incidence of adverse reactions associated with IT. It also improved QoL for patients and their parents.

Omalizumab already has its established place in EAACI (European Academy of Allergy and Clinical Immunology) Guidelines on management of IgE-mediated food allergy and AAAAI (American Academy of Allergy, Asthma & Immunology) consensus-based guidelines on of omalizumab as food allergy treatment.^21^^,^^22^ According to the World Allergy Organization (WAO) manifesto on biologic therapies for food allergy (2025), the use of omalizumab is highly recommended for the treatment of food allergies, with the aim of minimizing food-related anaphylaxis. The authors also call for the establishment and promotion of a unified algorithm for the treatment of food allergies on a global scale.^23^

Research on larger groups of patients, preferably in the form of randomized clinical trials, should include the establishment of a unified schedule of omalizumab use in this indication, identification of a group of patients who will benefit most from such treatment, and evaluation of the long-acting effects of OMA on the immune system. A selection of studies focusing on this particular indication are presented in Table 3.

**Table 3:** The use of omalizumab during specific immunotherapy with food allergens.

Authors	Studied food	Number of respondents	Type of study	OMA Dosage, OIT Regimen	Conclusions
MacGinnitie, A. J. et al.^61^	Peanuts	N = 36	DBRPCT phase 2	OMA for 12 weeks; 1-day DS up to 250 mg; weekly dose escalation to 2000 mg	OMA allowed for an increase in the tolerated allergen dose (10x) on the day of initial DS. 6 weeks after the last dose of OMA, 85% tolerated 2000 mg of peanut vs. 12.5% in the placebo group.
Yoosefi Moridani et al.^62^	Peanut	N = 3 (children)	Case series	OMA for 8 weeks (300 mg every 4 weeks) at week 12 OMA + OIT (initiation dose), continuation of OMA + OIT every 4 weeks	After OMA pretreatment patients with initial anaphylaxis to peanut protein successfully underwent initial phase of OIT and reached maintenance dose without anaphylaxis.
Wood R. A. et al.^16^	Cow's milk	N = 57	DBRPCT	OMA for 28 months; MOIT started in the 4th month of the trial; OFC at month 28-th and continuation of OIT for another 8 weeks	OMA reduced the incidence of adverse reactions during MOIT dose escalation (2.1% vs 16.1%; p = 0.0005) and treatment-requiring responses (0.0% vs 3.8%, p = 0.0008), and reduced the total number of doses to maintain tolerance to the allergen (198 vs 225; p = 0.008).
Giavina-Bianchi et al.^63^	Cow's milk	N = 1 (17y.o. M)	Case report	OMA 600 mg every 2 weeks (according to IgE and weight) for 6 months next 2-day rapid desensitization up to 65 ml/day maintenance dose	After pretreatment with OMA patient with history of anaphylaxis in contact with cow's milk was able to underwent desensitization without severe adverse effects (mild skin symptoms only).
Andorf S. et al.^64^	Multiple foods	N = 48	DBRPCT phase 2	OMA for 16 weeks; OIT started at week 8; OFC at week 36.	More effective DS (OR 10.0, 95% CI 1.8–58.3, p = 0.0044) and fewer adverse events at dose escalation (27% *vs* 68%; p = 0.0082) due to the addition of OMA.
Wood R.A. et al.^65^	Multiple foods	N = 177	DBRPCT phase 3	OMA every 2 or 4 weeks at a dose according to weight and IgE level (continued for 16–20 weeks)	OMA vs placebo improved tolerance of allergenic foods: 67% vs. 7% for peanut, 41% vs. 3% for cashew, 66% vs. 10% for cow's milk; 67% vs. 0% for chicken egg (p < 0.001).
Zuberbier T. et al.^8^	Multiple foods	N = 953	Meta-analysis 36 studies (9 RCTs, 19 CCTs and 8 observational studies)	–	OMA significantly increases the tolerated dose of allergens and improves QoL; It provides faster DS and a higher tolerated dose in the OIT.
Nurmatov et al.^20^	Multiple foods	N = 1010	Meta-analysis 15 studies (13 RCTs, 2 NCTs)	–	OMA improved desensitization (RR 2.04), increased food tolerance (RR 4.90), reduced FA reaction risk (RR 0.55) without increased AEs; decreased hypersensitivity/inflammation; improved QoL (patients & parents); no cost-effectiveness data.
Alexiou et al.^66^	Multiple foods	N = 62	Retrospective analysis	OMA + OIT vs. OMA (mostly 300 mg every 4 weeks regimen)	Among 83,9% treatment respondents, 9,7% achieved DS, 9,7% had decrease in allergy symptoms and 64,5% presented no anaphylactic reactions during treatment.

Abbreviations: DBRPCT, double-blind randomized placebo-controlled trial; OMA, omalizumab; DS, desensitization; MOIT, milk oral immunotherapy; OFC, oral food challenge; OR, odds ratio; CI, confidence interval; RCT, randomized controlled trial; CCT, controlled clinical trial; QoL, quality of life; NCT, National Clinical Trial; FA, food allergy, AE, adverse event

It is important to acknowledge that, in contrast to other off-label indications, omalizumab has been demonstrated to be effective in this particular indication through the utilisation of numerous randomised controlled trials and meta-analyses mentioned above.

### Drug hypersensitivity

Drug hypersensitivity is a heterogeneous group of diseases characterized by different mechanisms and clinical symptoms. The most common causes of those reactions are antibiotics and non-steroidal anti-inflammatory drugs (NSAIDs). To the best of our knowledge, no studies have analyzed the use of OMA in hypersensitivity to antibiotics (perhaps due to the large number of alternative drugs). The available studies have mainly focused on the efficacy of omalizumab in hypersensitivity to NSAIDs and anticancer drugs.

Omalizumab was considered relatively early in the treatment of N-ERD (respiratory disease exacerbated by NSAIDs). In patients with this disease, the coexistence of chronic sinusitis with nasal polyps, bronchial asthma, and hypersensitivity to NSAIDs is observed, which manifests mainly as respiratory symptoms. The potential mechanism of action of omalizumab in N-ERD appears to be independent of direct IgE inhibition, and may depend on a reduction in mast cell excitability, which translates into a reduction in the production of pro-inflammatory lipid mediators. It has also been suggested that OMA may reduce the number of eosinophils in tissues and peripheral blood. Studies in patients with N-ERD indicate that OMA treatment has a positive effect on the course of bronchial asthma and reduces the need for SABA (short-acting beta-2 agonists) and oral glucocorticosteroids (oGCs). However, greater therapeutic effectiveness has been observed in patients with concomitant atopy.^24^ Omalizumab has been approved for the treatment of chronic rhinosinusitis with nasal polyps (CRSwNP), and a post-hoc analysis of POLYP1, POLYP2, and OLE registration studies have shown that its efficacy is independent of atopy, peripheral eosinophilia, or asthma.^25^ OMA may also have a beneficial effect in patients with N-ERD by reducing the severity of CRSwNP within 9 months of therapy.^26^ In addition, using omalizumab prior to the provocation test reduced the symptoms of the acute phase induced by aspirin intake. This is associated with reduced urinary leukotriene production.^27^ Similarly, treatment with OMA prior to ASA desensitization in patients with N-ERD with concomitant atopy resulted in fewer respiratory side effects during desensitization.^28^ However, few studies comparing the efficacy of omalizumab in improving asthma control and CRSwNP have indicated that hypersensitivity to NSAIDs is not a predictor of a better response to OMA.^29^

Hypersensitivity to drugs used in oncology is a special situation because life-threatening conditions and a lack of alternatives may lead to the decision to desensitize the drug. This is also related to the fact that premedication (consisting of glucocorticoids, antihistamines, and others) often has an insufficient effect, and switching to an anticancer drug is not always possible due to a divergent spectrum of action or cross-reactivity. Only a few immediate reactions to chemotherapeutic agents are IgE mediated. This mechanism is suspected to be responsible for allergies to platinum derivatives and, less frequently, to other drugs, such as taxanes and l-asparaginase. However, existing case reports and case series support the efficacy of OMA in IgE-independent response cases. In 1 series of cases, patients who had previously experienced a severe allergic reaction (with hemodynamic instability) to a selected anticancer drug (platinum derivatives, docetaxel, and rituximab) were able to continue therapy after OMA treatment with only minor side effects (mainly skin-related). Omalizumab effectively prevented immediate and delayed allergic reactions. In addition, OMA demonstrated efficacy in the mixed phenotype of oxyplatin hypersensitivity (simultaneous occurrence of IgE-mediated and cytokine mechanisms). A better desensitization effect was observed with subsequent doses of omalizumab.^30^

The authors of the study suggest administering 300 mg of OMA prior to desensitization with a follow-up dose every 2–3 weeks depending on the chemotherapeutic cycle (if hypersensitivity symptoms persist). Subsequent OMA administration periods may be extended every 4 weeks if desensitization is well tolerated.^28^

There are isolated case reports concerning the beneficial role of OMA in insulin desensitization in patients with diabetes.^31^

Some authors indicate that omalizumab may be part of managing Severe Cutaneous Adverse Reactions (SCARs) such as Drug Reaction with Eosinophilia and Systemic Symptoms (DRESS)^32^ and Toxic epidermal Necrolysis (TEN).^33^ A limited number of clinical cases have been documented, and while some treatment outcomes have been reported, the abrupt nature of these diseases, the necessity to rapidly neutralise activated immune cells (eosinophils in DRESS; cytotoxic T lymphocytes, NK cells in TEN), and the fact that omalizumab lacks direct cell-reducing properties, suggest that alternative medications may be more appropriate for these indications. These include corticosteroids, cyclosporine, IVIG, and topical treatments.^34^^,^^35^

### Other selected diseases in which OMA has proven effective

For allergic bronchopulmonary aspergillosis, data from systematic reviews and meta-analyses have indicated a reduction in the risk of exacerbations of the disease, a reduction in the use of oral glucocorticoids (OCS), and improved asthma control.^36^ In patients with systemic mastocytosis, an observational study confirmed a reduction in the incidence of recurrent episodes of anaphylaxis and severity of skin symptoms characteristic of this disease.^37^

A post-hoc analysis of the STELLAIR study (a retrospective real-world study) of patients with allergic asthma and at least 3 concomitant allergic diseases showed that these patients benefited more clinically from treatment with omalizumab than those with fewer comorbidities.^38^ In the context of allergic eye diseases, observational studies have shown a reduction in the severity of conjunctivitis symptoms (assessed using the Ocular Severity Index) and an improvement in quality of life (QoL).^39^

In a retrospective study of patients with bullous pemphigoid, 77% achieved complete remission and 9% achieved partial remission. The mean time to remission was 3 months, while symptom control was achieved within 10 days.^40^ In the case of idiopathic angioedema, in a randomized clinical trial in adults (N = 10) who had experienced at least 2 episodes of angioedema in the last 6 months without an apparent laboratory or clinical cause, the results showed an improvement in the Angioedema Activity Score (AAS) and a reduction in the number of episodes per month.^41^ In the course of allergic rhinitis, data from meta-analyses and randomized trials have indicated an improvement in the Daily Nasal Rescue Medication Score (DNRMS), a reduction in the use of antihistamine drugs, and an improvement in the quality of life^42^^,^^43^.

In addition to those mentioned above, there are also reports, mainly in the form of case reports and pilot studies, of the potential efficacy of OMA in the treatment of eosinophilic gastritis and gluten-dependent enteropathy,^44^ eosinophilic otitis media,^45^ exercise-induced anaphylaxis,^46^ and idiopathic anaphylaxis.^47^

### Further research directions

Among further areas of research, there is still a significant need to learn more about the mechanisms of action of omalizumab, particularly those not directly related to IgE binding. In addition, further safety monitoring is crucial, considering vulnerable populations, such as pregnant women. Further analysis is needed to clearly determine the risks associated with the long-term use of this drug. In terms of safety, it is important to clarify the role of omalizumab antibodies (ADA).^48^ Another area of research is cost-effectiveness analysis. Currently, these studies have only been conducted in patients with severe asthma. Despite the high cost of therapy, they indicated that treating severe asthma with omalizumab is ultimately cost-effective, as it reduces exacerbation frequency, improves disease control, and enhances quality of life^49^^,^^50^ The cost-effectiveness of omalizumab therapy for other registered and off-label indications, that is, the relationship between the costs and health effects (eg, improvement of quality of life, reduction of symptoms), has not yet been sufficiently analyzed. Based on the available data, it can be assumed that given the relatively high cost of OMA therapy, candidates for treatment outside the registered indications should be carefully selected. In the above disease entities, the risk of hospitalization is much lower than in asthma, affecting only a small percentage of patients if it does occur. Therefore, OMA therapy should be considered for selected patients who do not respond to previous treatment or show significant disease progression, considering the clinical benefits and available resources.

Further research should also include the identification of factors and biomarkers that would predict the response to OMA, especially in existing indications. Such an approach will allow for a more precise personalization of treatment, minimization of adverse effects, and effective monitoring of the effects of therapy and the course of the disease. An interesting aspect is the explanation for the legitimacy of omalizumab use in patients with several concomitant allergic diseases. Previous studies have indicated that in patients with asthma and other allergic diseases, the effectiveness of OMA does not decrease with an increase in the number of comorbidities; therefore,^51^ it is possible to simultaneously treat several diseases with 1 active agent.

Currently, there is no standardization of the dosage or duration of OMA therapy. In bronchial asthma, dosage is usually determined according to serum IgE levels and body weight. In chronic spontaneous urticaria, the dose may be determined on an individual basis depending on the response to treatment, starting with the administration of 300 mg OMA every 4 weeks. In other indications, the use of an OMA is usually assumed in a regimen similar to that in spontaneous urticaria.

There are reports that OMA may have a long-term suspended effect on IgE production,^52^ which could justify the discontinuation of treatment after several years. However, the issue of remission of IgE-mediated diseases has not been fully explained. Future studies should assess sustained unresponsiveness, that is, determine whether OMA's protective effect continues beyond the end of drug administration and whether it could act as a disease-modifying drug.

The creation of a centralised registry to monitor long-term outcomes and treatment adherence has the potential to enhance understanding of omalizumab-facilitated OIT in real-world practice.^53^

## Author contributions

AJ performed the literature search and data extraction and drafted the initial manuscript. AW conceived the idea for the review, developed its concept and structure, and supervised the project. Both authors critically revised and approved the final version of the manuscript.

## Authors’ consent for publication

All authors have read and approved the final version of the manuscript and consent to its publication.

## Declaration of generative AI and AI-assisted technologies in the writing process

During the preparation of this work the authors used Paper Pal exclusively for language editing and grammar improvement. The tool was not used for generating, interpreting, or drawing scientific conclusions. After using this tool/service, the authors reviewed and edited the content as needed and take full responsibility for the content of the publication.

## Funding

This research received no external funding.

## Conflict of interest declaration

The authors declare that they have no conflict of interest related to this study.
